# Preparing Polypyrrole-Coated Stretchable Textile via Low-Temperature Interfacial Polymerization for Highly Sensitive Strain Sensor

**DOI:** 10.3390/mi10110788

**Published:** 2019-11-17

**Authors:** Xiaodie Chen, Bintian Li, Yan Qiao, Zhisong Lu

**Affiliations:** 1 Key Laboratory of Luminescent and Real-Time Analytical Chemistry (Southwest University), Ministry of Education, School of Materials & Energy, Southwest University, Chongqing 400715, China; 2 Institute for Clean Energy & Advanced Materials, School of Materials & Energy, Southwest University, Chongqing 400715, China

**Keywords:** textile, strain sensor, low-temperature interfacial polymerization, wearable electronics, human motion

## Abstract

The stretchable sensor has been considered as the most important component in a wearable device. However, it is still a great challenge to develop a highly sensitive textile-based strain sensor with good flexibility, excellent skin affinity, and large dynamic range. Herein, polypyrrole (PPy) was immobilized on a stretchable textile knitted by polyester and spandex via low-temperature interfacial polymerization to prepare a conductive strain sensor for human motion and respiration measurements. Scanning electron microscopy, Fourier transform infrared spectrometry, and thermal gravimetric data verify that a thin layer of PPy has been successfully coated on the textile with a high density and very uniform distribution. The resistance of the as-prepared textile is 21.25 Ω/cm^2^ and the PPy-coated textile could be used as an electric conductor to light up a LED lamp. Moreover, the textile could tolerate folding at an angle of 180° and 500 times of bending-twisting cycles without significant changes on its resistance. A negative correlation between the resistance change and the applied strain is observed for the textile-based sensor in the strain ranging from 0 to 71% with the gauge factor of −0.46. After more than 200 cycles of stretching-releasing under the strain of 26%, there is no obvious alteration on the sensing responses. The sensors were attached on volunteers’ body or clothes for the real-time measurement of human motions and respiration, demonstrating that the textile-based sensor could sensitively detect finger, elbow, and knee bending and differentiate deep, normal, and fast breath. This work may provide an approach to uniform and dense coating conductive polymers on textiles for highly sensitive and stretchable sensors, which possess great potentials in practical applications for real-time monitoring human motions and physiological signs.

## 1. Introduction

As advances of flexible and stretchable electronics, various wearable devices have been developed in the past decades to meet people’s requirements on real-time fitness tracking, daily health monitoring, and early disease diagnostics [1,2]. Strain sensors have been considered as crucial building blocks for wearable devices due to their great capability in sensing slight changes of human body and human skin [3]. After being directly attached to the clothes or skin, they could precisely detect a wide range of human motions and physical signs like respiration rate and heart rate [2,3,4,5]. Therefore, it is of great demand to design highly sensitive strain sensors with good flexibility, excellent skin affinity, and large dynamic range [4].

Polymers have been extensively investigated as the matrix to composite with conductive materials for the fabrication of stretchable strain sensors [6]. Polyurethane and poly(3,4-ethylenedioxythiophene):poly(styrenesulfonate) have been blended together to construct a polymeric composite-based flexible strain sensor [7]. A patch-type strain sensor with both high stretchability and sensitivity has been fabricated by simple dropping conductive gold nanowires on a stretchable latex rubber [8]. A highly sensitive strain sensor with the stretchability up to 100% has also been developed based on piezoresistive graphene-nanocellulose nanopaper [9]. Although the polymers-based sensors are easy-to-use and very flexible, their skin affinity and air/moisture penetrability are still great concerns to the end users. A textile is a flexible material consisting of a network of fibers, which has been utilized for wearing over human history. Due to its light-weight, mass production, and superior flexibility [5], the textile has been considered as an ideal material for the preparation of wearable strain sensors [10]. In comparison to the conventional polymeric film, the woven structure of the textiles allows the free exchange of air and moisture between the environment and human skin, which is quite important for the long-term usage of the skin-attached wearable devices. In the past few years, extensive efforts have been dedicated to enable the textile with conductivity for the fabrication of stretchable strain sensors [4,5,8,11]. In the early studies, metal nanoparticles were in situ grown on the textile surface to form the conductive layers [8,12,13]. This method could provide good conductivity for the functionalized textiles at the relax state. However, the metal coating layer could be damaged under the strain, further resulting in the sharp drop of the conductivity. Conductive nanomaterials, such as silver nanowires and carbon nanotubes, have been dispensed on the textile substrates to form conductive coatings [14,15]. The inter-crosslinked nanowires or nanotubes could tolerate the stretching, but the high cost of the conductive materials and the complicated fabrication procedures may hinder their mass-production and feasibility.

Conducting polymers are a class of electronic materials with excellent processability, good stability, and high electric conductivity [16]. As a typical representative of conducting polymers, polypyrrole (PPy) has been applied in a wide range of fields including battery [17,18,19], supercapacitors [20,21], sensors [22,23], ion-sieving [24], corrosion protection [25,26], microwave shielding [27,28,29], e-textiles [5], and artificial muscles [10] due to its high conductivity, good environmental stability, low-cost, good adhesion, and non-toxicity [30]. PPy-coated Lycra, cotton spandex, and cotton have been manufactured as textile-based strain sensors via in situ monomer vapor deposition [31], electrochemical polymerization [24], and in situ oxidative chemical polymerization [11]. The in situ monomer vapor deposition method is quite tedious and needs expensive instruments like screen-printer. The electrochemical polymerization could only be adapted to the conductive textiles. As to the in situ oxidative chemical polymerization, the high reaction rate may lead to the non-uniform distribution and aggregation of PPy on the fiber surface. Low-temperature interfacial polymerization is a facile, economical, and efficient method to produce uniform PPy thin film at the interface of two phases with a relatively low reaction rate [32]. It has been conducted to prepare paper- and textile-based supercapacitors for the achievement of high capacitances [21,32]. As far as we know, low-temperature interfacial polymerization has not been used to immobilize PPy on a stretchable textile for the fabrication of textile-based strain sensors.

In this work, PPy was coated on a stretchable textile knitted by 92% polyester and 8% spandex via low-temperature interfacial polymerization to prepare a conductive strain sensor for human motion and respiration monitoring. Scanning electron microscopy (SEM), Fourier transform infrared spectrometry (FTIR), and thermal gravimetric analyzer (TGA) were carried out to characterize the pristine and PPy-modified polyester/spandex textiles. Effects of the bending angle and 500 times of bending/twisting on the resistance of the PPy-coated textiles were also measured to investigate the flexibility and stability. The sensing performance of the PPy-modified textiles was studied with the strain rising from 0 to 100%. The sensing mechanism was discussed based on the optical images of a sensor at its releasing and stretching states. Finally, the as-prepared sensors were fixed on finger, elbow, knee, and abdomen of the volunteers to demonstrate their potentials in practical monitoring of human motion and respiration.

## 2. Materials and Methods 

### 2.1. Materials

Pyrrole (99%) was purchased from Adamas-beta Co. (Shanghai, China). Iron chloride hexahydrate (FeCl_3_∙6H_2_O), cyclohexane, and p-toluenesulfonic acid (PTS) were bought from Aladdin Reagent Co. (Shanghai, China). All other chemicals were of analytical grade and directly used without further purification. Deionized water was produced by a Millipore Q water purification system. The polyester/spandex-based stretchable textile was fabricated by BJD Clothes Co. (Zhengjiang, China). Before the experiments, the polyester/spandex textile was cut into small pieces with the desired shapes and sizes to meet the requirements of human motion and respiration measurements.

### 2.2. Preparation of PPy-Coated Textiles

**Figure 1:**
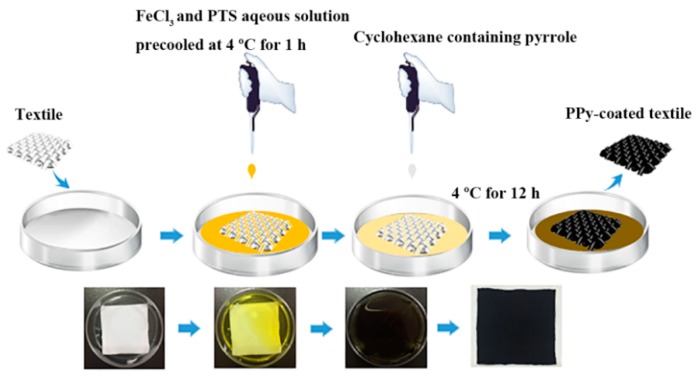
Preparation of polypyrrole (PPy)-coated textile via low-temperature interfacial polymerization.

The PPy-functionalized textile was prepared according to Chen’s approach with minor modifications [32] (Figure 1). Firstly, 5 mL of 0.4 M FeCl_3_∙6H_2_O and 4 mL of 0.4 M PTS were mixed in a petri dish, followed by pre-cooling at 4 °C for 1 hour. A polyester/spandex-based stretchable textile with the size of 40 by 40 mm^2^ was immersed into the above mixture. Then, 9 mL cyclohexane containing 150 μL pyrrole was slowly dropped into the FeCl_3_-PTS aqueous solution. Due to the immiscibility of water and cyclohexane, two phases were formed in the petri dish with the organic phase on top. The whole reaction system was stored at 4 °C for 12 hours. During this process, the color of the textile gradually changed from white to black. The functionalized textile was collected and thoroughly rinsed with water for three times to remove free polymers. Then, the textile was dried at 40 °C for 1 hour. The above procedures were repeated one more time to immobilize a dense PPy layer on the textile.

### 2.3. Characterization

Surface morphologies of the polyester/spandex textiles before and after PPy modification were characterized using a JSM-7600 FESEM (JEOL, Tokyo, Japan). A smartphone-attached optical microscope (Wellwa, Guangdong, China) was applied to image the structural change of the PPy-coated textile under tension at micrometer scale. The surface functional groups and the thermal stability of the textile before and after PPy coating were examined with a Fourier transform infrared spectrometer (FTIR, Nicolet 6700, Thermo Electronic Corporation, Waltham, MA, USA) and a SDT-Q600 thermogravimetric analyzer (TA Instruments, New Castle, DE, USA), respectively. 

### 2.4. Strain Sensing Tests

The resistances of the PPy-coated polyester/spandex textiles were measured using a Keithley 2400 Source Meter. A 20 mm by 40 mm textile was bent at an angle of 0–180° for the measurement of its resistance alteration. The resistance of the same textile was also examined after 500 times of bending or twisting to investigate the stability of the PPy layer. The textiles with the size of 20 mm by 40 mm were stretched with an electronic universal testing machine (WDW-211, Jinan, China) at the velocity of 500 mm/min to achieve the designed strains. Strain-induced resistance changes were collected to explore the strain sensing range.

### 2.5. On-Body Tests

The PPy-coated polyester/spandex textiles shaped as per the requirements of on-body measurements were fixed on clothes or skin to demonstrate their potentials in practical applications. The sensing performance of the device were collected with an electrochemical workstation (CHI 660E, CH Instrument Company, Shanghai, China). The current changes induced by the bending of finger, elbow, and knee were measured with the PPy-coated textile at an operating voltage of 0.1 V. The textile-based sensor was also attached on the volunteer’s abdomen to sense the up and down movement caused by human breathing. The current patterns were collected to track deep breath, fast breath, and normal breath.

## 3. Results and Discussion

**Figure 2:**
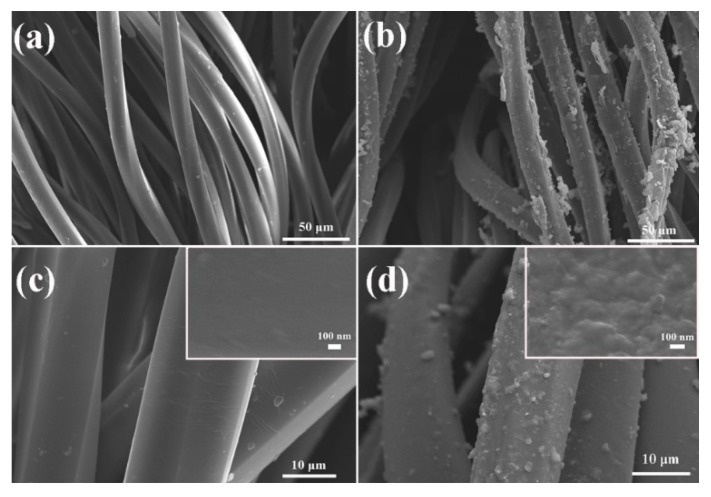
SEM images of pristine polyester/spandex textile at low (**a**) and high magnifications (**c**); SEM images of PPy-coated polyester/spandex textile at low (**b**) and high magnifications (**d**); and inset in (**c**,**d**): Detailed surface morphologies of the fibers before and after PPy coating, respectively.

SEM was employed to characterize surface morphologies of polyester/spandex textiles before and after PPy immobilization. As shown in Figure 2a,c, the pristine polyester/spandex textile is woven with fibers, which possess very smooth surface and the diameter of ~20 μm. After low-temperature interface polymerization, the fiber surface became rough (Figure 2b) and a lot of small particles could be observed (Figure 2d). High-magnified image (Inset of Figure 2d) further illustrates that the fiber is fully covered with a dense and uniform film, which is significantly different from the loosely attached particles prepared with chemical oxidative polymerization at room temperature [33]. The greatly reduced reaction rate and the polymerization occurred at the interface between two phases may cause the formation of the uniform and thin PPy layer. The data strongly proves that PPy has been successfully immobilized on the textile surface to function as a continuous conductive layer. The same approach could also be applied to successfully immobilize PPy on nylon/lycra, silk, and cotton textiles. Their maximum strains much lower than that of PPy-coated polyester/spandex textile. Thus, we chose polyester/spandex textile as the substrate to prepare the strain sensor in this work (Figures S1 and S2 in supporting information).

**Figure 3:**
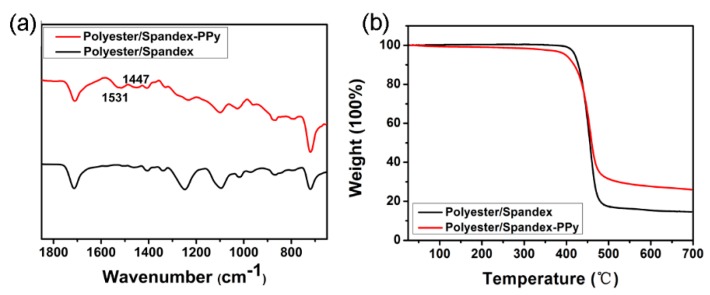
(**a**) FTIR spectra of the polyester/spandex textiles before and after PPy modification. (**b**) thermal gravimetric analyzer (TGA) curves of the polyester/spandex textiles before and after PPy modification in an atmosphere of nitrogen.

FTIR spectra were collected to further verify the existence of PPy on the textiles. In comparison to pristine polyester/spandex textile, two weak peaks appear at 1447 and 1531 cm^−1^ in the spectra of the PPy-coated one (Figure 3a). The former peak could be attributed to the C=C and C-C stretching vibration of PPy ring [32]. The latter one may be ascribed to the pyrrole ring characteristic vibration [34]. The thermal stability of the textiles was also investigated by measuring the weight change in the temperature range of 25–700 °C under the nitrogen atmosphere. It can be seen from Figure 3b that the weight of the pristine textile keeps at a very stable level as temperature increases from 25 to 400 °C. However, after PPy coating, the sample undergoes a gradual weight loss of ~5%, which may be due to the breakdown of the PPy backbone and chemical decomposition of the dopant FeCl_3_. Interestingly, the remaining weights of pristine and PPy-modified textiles at 700 °C are 14.6% and 25.0%, respectively. The relatively high content of carbon element in PPy may lead to the remarkable enhancement on the residue weight of PPy-modified sample. The FTIR and TGA data also verify that the PPy has been immobilized on the textiles, agreeing well with the SEM images.

**Figure 4:**
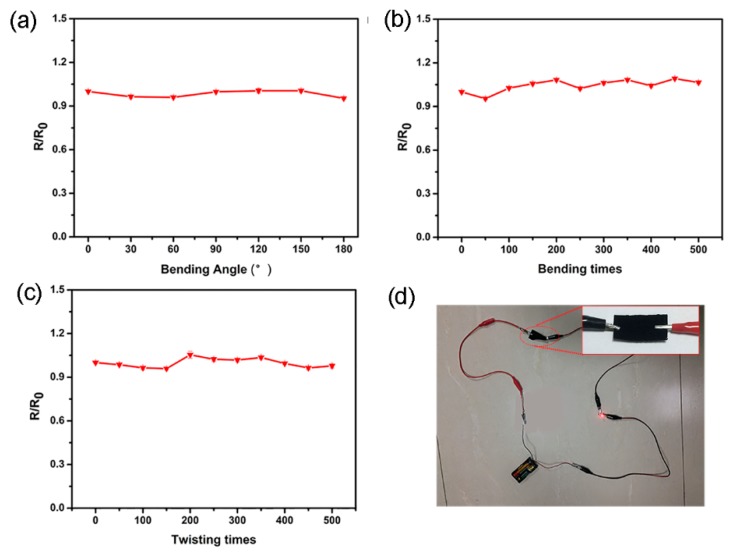
(**a**) The normalized resistance of PPy-coated polyester/spandex textile at the bending angle of 0–180°; effects of repeated bending (**b**) and twisting (**c**) on the normalized resistance of PPy-coated polyester/spandex textiles; and (**d**) demonstration of the conductivity of PPy-coated polyester/spandex textile.

Before the sensing tests, the resistance of the PPy-coated polyester/spandex textile is measured as 21.25 Ω/cm^2^, which is comparable to the PPy-modified polyester/spandex and cotton/spandex fabrics [35]. We changed the pyrrole volume in the polymerization system to control the amount of PPy coated on the textiles. As the monomer volume rises from 50 to 150 μL, the amount of PPy coated on the textiles gradually increases. When the volume further enhances to 200 μL, a slight reduction on the PPy amount is observed (Figure S3a in supporting information). Moreover, the amount of surface-coated PPy could significantly affect the conductivity of the textile. Large amount of PPy on the textile may lead to a high level of conductivity (Figure S3b in supporting information). Since the conductive textile is designed as a strain sensor, we must rule out the interferences of bending and twisting on the textile resistance. Thus, we investigated the effects of bending angle and repeated bending/twisting on the normalized resistance of the PPy-functionalized textile. When the textile was bent from 0° to 180°, the resistance remains at a constant level, suggesting that the bending does not affect its electronic property. Since bending and twisting cannot be avoided during the practical applications of wearable devices, the influences of repeated bending and twisting on the textile resistance were also studied. As shown in Figure 4b,c, the normalized resistance is also stable during 500 times of twisting and bending, indicating that the textile can tolerate repeated bending and twisting without alteration on the device conductivity. To demonstrate the conductivity, a PPy-immobilized textile was connected with a LED and two batteries in series. The textile could function as an electrical conductor to light up a LED lamp with lowest working voltage of 2.0 V (Figure 4d).

**Figure 5:**
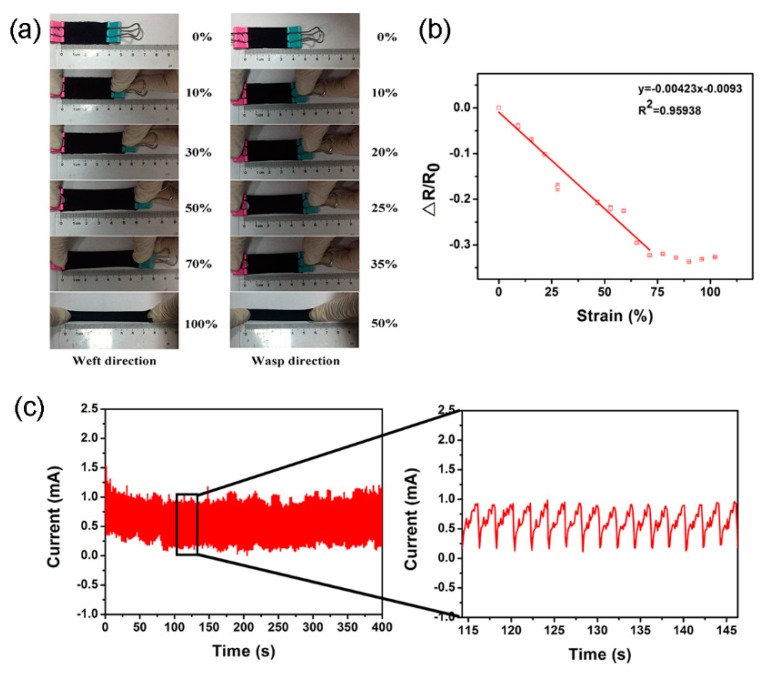
(**a**) PPy-coated polyester/spandex textiles stretched to different strain ratios along the weft and warp directions; (**b**) relative resistance change (Δ*R*/*R*_0_) as a function of tensile strain; and (**c**) current responses of a textile-based sensor upon periodic stretching-releasing cycles under a strain of 26%.

The tensile property of the PPy-coated polyester/spandex textile is quite different in the wasp and weft directions. Along the weft direction, the textile can be stretched up to the strain of 100%, which is ~2 times higher than the maximum strain in the wasp direction (Figure 5a). Therefore, in the following experiments, the stretching force was applied along the weft direction to investigate the properties of the textile-based strain sensor. The gauge factor (GF) is employed to evaluate the tensile sensitivity according to the following equation:

GF = (Δ*R*/*R*_0_)/*ε* (1)

where Δ*R* and *R*_0_ are the change of resistance and the initial resistance, respectively, and *ɛ* is the strain applied in the test. As illustrated in Figure 5b, the normalized resistance change is negatively correlated to the strain applied to the PPy-coated textile in the strain range of 0 to 71% with *R*^2^ of 0.96 (Figure 5b). The GF is calculated to be −0.46 in the linear sensing range (0–71%). The performance of the textile-based strain sensor could be prominently affected by the amount of PPy coating layer (Figure S3 in supporting information). When the mass ratios of PPy on the textiles are 0.128 and 0.180, the stretching of the sensors induce the enhancement of the textile resistance. On the contrary, the resistance of the sensor decreases at the stretching state as the mass ratio increases to 0.183. It can be clearly seen that the sensor with the PPy mass ratio of 0.183, which was prepared at the monomer volume of 150 μL, has the largest absolute value of GF and the widest sensing range. The durability of the PPy-coated polyester/spandex sensor is studied under stretching-releasing cycles with the strain of 26% and the applied voltage of 0.1 V. It can be seen that the current responses to the repeated stretching and releasing keeps very stable even after 200 cycles (Figure 5c). Significant changes on the current profiles occur after 200 stretching-releasing cycles, indicating that the sensor can tolerate 200 times of stretching-releasing without alteration on its sensing performance (Figure S4 in supporting information). The non-uniform stretching velocity applied by the universal testing machine may lead to the zigzag current changes in Figure 5c. In comparison to the strain sensors based on PPy-coated textiles that were prepared with other approaches [11,22,31,36,37,38], the as-prepared one possesses the largest dynamic range as well as the comparable GF and conductivity (Table 1). More importantly, for the textile-based sensor, the resistance at the stretching state is lower than that at the releasing state. A much more significant current signal may be collected at a higher strain under a constant applied voltage, which is favorable for the practical applications.

**Figure 6:**
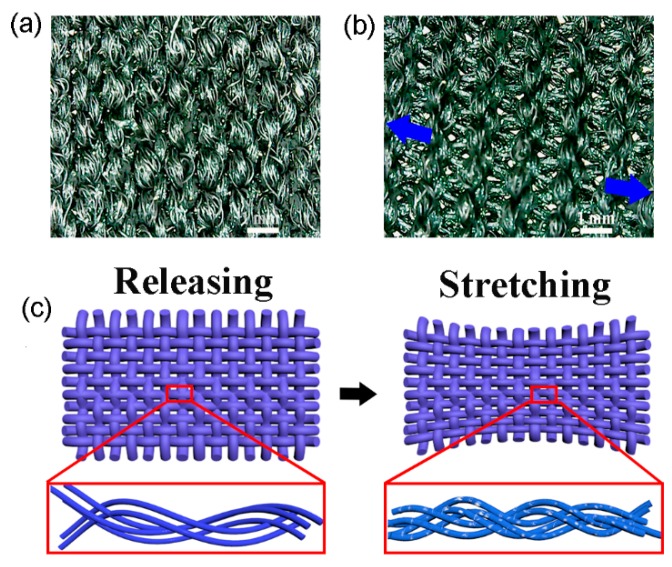
Microscopic images of a PPy-coated polyester/spandex textile at the releasing (**a**) and stretching (**b**) states. (**c**) schematic illustration of the structural change of the PPy-coated polyester/spandex textile under a stretching force. Arrows in (**b**) show the stretching direction.

To understand the sensing mechanism, a PPy-coated textile was examined using an optical microscope at both releasing and stretching states (Figure 6a,b). A typical woven structure could be observed in both images. At the releasing state, the fibers are loosely entangled together. When the strain is applied in the weft direction, the warp fibers are aligned and tightly hold together with each other. According to Holm’s contact resistance theory, higher pressure and more contact points lead to the lower contact resistance [38]. Since PPy has been coated on the fibers during the low-temperature polymerization process, the tight contacts of the fibers may significantly increase the pathways in the textiles for the electron transfer, thus reducing the resistance of the sensors (Figure 6c). However, the effects of strain on individual fibers should also be considered in the sensing mechanism. The application of a strain may lead to the rupture of the PPy coating layer, which obviously leads to the enhancement of the textile resistance. Therefore, the strain-induced addition of the electron transfer pathways and rupture of the conductive coating layers may both contribute to the performance of the textile-based sensors. As shown in Figure S3 (ESI), positive GFs were observed in the textiles with thin PPy layers because of the easy fraction of the conductive layers under tensile loading. While, negative GFs appear in the samples containing more PPy since the addition of contact points plays a prominent role.

**Figure 7:**
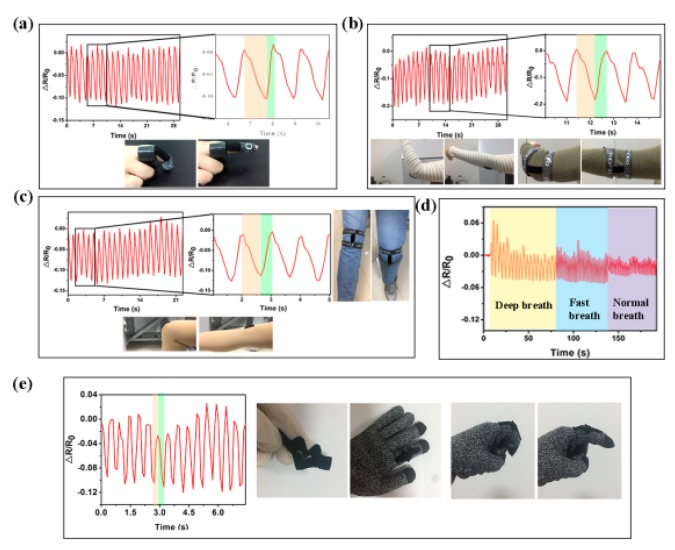
Responses of the textile-based strain sensors to human finger bending (**a**), elbow bending (**b**) and knee bending (**c**). Inserts are the photos showing the attachment of the devices on human body. (**d**) Real-time measurements of respiration by fixing a sensor on the volunteer’s abdomen. (**e**) Real-time measurements of finger bending by a glove-stitched sensor.

To further demonstrate the feasibility of the strain sensor based on PPy-coated polyester/spandex textiles, the devices were fixed on body to test their capability for human motion and respiration monitoring. As shown in Figure 7a–c, repeated bending of finger, elbow, and knee could be sensitively real-time detected with the as-prepared strain sensors. Once the volunteer bends the finger, elbow, and knee, stretching forces are applied to the sensors, resulting in the sharp reduction of the resistance. As the finger, elbow, and knee are straightened again, the resistances recover to the original levels. The PPy-coated textile was also stitched on the clothes to sense the breath-caused changes on the abdomen (Figure 7d). During the inhalation process, the abdomen contracts, leading to the rising of resistance. The abdomen relaxes in the exhalation process and the resistance decreases. The strain sensor can not only monitor the respiration rate, but also differentiate various breathing patterns like deep breath, normal breath, and fast breath. Inspired by Zhao and Shu’s work [39,40,41], to demonstrate its great compatibility with daily clothing, the textile-based sensor was stitched on a glove for the sensing of finger bending, also showing very sensitive responses (Figure 7e). Based on above results, it can be concluded that the PPy-coated polyester/spandex textile is very promising to be used as the strain sensor for real-time monitoring human motions and precise tracking human’s cardiopulmonary function.

## 4. Conclusions

In summary, we have prepared a PPy-coated stretchable textile via low-temperature interfacial polymerization method to function as a sensitive and wearable strain sensor for monitoring human motions and respiration. The PPy film is uniformly and densely deposited on the textile surface to produce a continuous conductive layer, rendering the as-prepared textile with the conductivity as high as 21.25 Ω/cm^2^. The as-prepared textile could be used as an electrical conductor to light up a LED lamp. More importantly, it can tolerate 500 times of bending and twisting without significant change on its resistance. The tensile tests show that the textile-based strain sensor possesses a negatively dynamic range from 0 to 71% with GF as −0.46. After more than 200 cycles of stretching-releasing under the strain of 26%, there is no obvious alteration on the sensing response. The on-body investigations further demonstrate that the sensor based on PPy-coated polyester/spandex textile could sensitively detect human motions like finger, elbow, and knee bending and differentiate human respiration patterns like deep, normal, and fast breath. This work may not only develop a strain sensor based on PPy-coated textile for practical applications in wearable electronics, but also provide an approach for the uniform and dense coating of conductive polymers on textiles.

## Figures and Tables

**Table 1:** Summary of previously reported strain sensors based on PPy-coated textiles.

Textile Types	Method	Resistance	Maximum Strain Range	Gauge Factor	References
Cotton spandex	In situ polymerization at 0 °C	3.83 Ω/cm^2^	0–9%	−1.23	[38]
Polyester/Spandex	In situ polymerization at 0 °C	6.77 Ω/cm^2^	0–8%	−0.05	[38]
Lycra	In situ polymerization at room temperature	375 Ω/cm^2^	0–60%	−0.69	[37]
Cotton/Spandex	In situ polymerization at 0 °C	8.93 Ω/cm^2^	0–25%	0.71(15−25%) 1.1(0−15%)	[22]
PU/PDMS	In situ polymerization at room temperature	130.63 Ω/cm^2^		0.15	[36]
Cotton	In situ polymerization at 0–8 °C	303 Ω sq^−1^	0–35%	0.35 (0–15%) 2.39(15−35%)	[11]
Nylon/Lycra	Screen-printing/vapor		0–20%	8	[31]
Polyester/Spandex	Low-temperature interfacial polymerization	21.25 Ω/cm^2^	0–71%	−0.46	This study

## Supplementary Materials

The following are available online at https://www.mdpi.com/2072-666X/10/11/788/s1, Figure S1: SEM images of pristine nylon/lycra, silk and cotton fabrics at low (a,e,i) and high magnifications (c,g,k); SEM images of PPy-caoted nylon/lycra, silk and cotton fabrics at low (b,f,j) and high magnifications (d,h,l). Figure S2: PPy-coated polyester/spandex, nylon/lycra, silk and cotton textiles stretched to the maximum strain. Figure S3: Mass ratios (a) of surface-coated PPy to the textile and resistance ratios (b) of the as-prepared conducting textiles after polymerization with different volume of Py. Relative resistance change (ΔR/R_0_) of the as-prepared textiles prepared with 50 μL Py (c), 100 μL Py (d), 150 μL Py (e) and 200 μL Py (f). Figure S4: Current responses of a textile-based sensor upon periodic stretching-releasing cycles under a strain of 26%.

## References

[1] Sekitani T., Someya T. (2010). Stretchable, large-area organic electronics. Adv. Mater..

[2] Zang Y.P., Zhang F.J., Di C.A., Zhu D.B. (2015). Advances of flexible pressure sensors toward artificial intelligence and health care applications. Mater. Horiz..

[3] Trung T.Q., Lee N.E. (2016). Flexible and stretchable physical sensor integrated platforms for wearable human-activity monitoring and personal healthcare. Adv. Mater..

[4] Cho J.H., Ha S.H., Kim J.M. (2018). Transparent and stretchable strain sensors based on metal nanowire microgrids for human motion monitoring. Nanotechnology.

[5] Chen S., Liu S.Q., Wang P.P., Liu H.Z., Liu L. (2018). Highly stretchable fiber-shaped e-textiles for strain/pressure sensing, full-range human motions detection, health monitoring, and 2D force mapping. J. Mater. Sci..

[6] Sun Q., Seung W., Kim B.J., Seo S., Kim S.W., Cho J.H. (2015). Active matrix electronic skin strain sensor based on piezopotential-powered graphene transistors. Adv. Mater..

[7] Seyedin M.Z., Razal J.M., Innis P.C., Wallace G.G. (2014). Strain-responsive polyurethane/PEDOT: PSS elastomeric composite fibers with high electrical conductivity. Adv. Funct. Mater..

[8] Gong S., Lai D.T.H., Su B., Si K.J., Ma Z., Yap L.W., Guo P.Z., Cheng W.L. (2015). Highly stretchy black gold E-skin nanopatches as highly sensitive wearable biomedical sensors. Adv. Electron. Mater..

[9] Yan C.Y., Wang J.X., Kang W.B., Cui M.Q., Wang X., Foo C.Y., Chee K.J., Lee P.S. (2014). Highly stretchable piezoresistive graphene-nanocellulose nanopaper for strain sensors. Adv. Mater..

[10] Ge J., Sun L., Zhang F.R., Zhang Y., Shi L.A., Zhao H.Y., Zhu H.W., Jiang H.L., Yu S.H. (2016). A stretchable electronic fabric artificial skin with pressure-, lateral strain-, and flexion-sensitive properties. Adv. Mater..

[11] Hao D.D., Xu B., Cai Z.S. (2018). Polypyrrole coated knitted fabric for robust wearable sensor and heater. J. Mater. Sci. Mater. El..

[12] Geng J., Zhu G., Yuan Y.K., Han G.Z. (2020). pH-mediated synthesis and mechanistic study of homogeneous magnetic Ag@Fe_3_O_4_ nanoparticles. J. Nanosci. Nanotechnol..

[13] Orouji A., Abbasi-Moayed S., Hormozi-Nezhad M.R. (2019). ThThnated Development of a pH assisted AgNP-based colorimetric sensor array for simultaneous identification of phosalone and azinphosmethyl pesticides. Spectrochim. Acta.

[14] Gao Y., Wang K., Song H.Z., Wu H., Yan S.C., Xu X., Shi Y. (2020). Fabrication and electrical properties of silver telluride nanowires. J. Nanosci. Nanotechnol..

[15] Dovjuu O., Kim S., Lee A., Kim J., Noh J., Huh S., Choi B., Jeong H. (2020). A simple approach for heat transfer enhancement of carbon nanofluids in aqueous media. J. Nanosci. Nanotechnol..

[16] Merlini C., Ramoa S.D.A.S., Barra G.M.O. (2013). Conducting polypyrrole-coated banana fiber composites: Preparation and characterization. Polym. Compos..

[17] Fedorkova A., Nacher-Alejos A., Gomez-Romero P., Orinakova R., Kaniansky D. (2010). Structural and electrochemical studies of PPy/PEG-LiFePO_4_ cathode material for Li-ion batteries. Electrochim. Acta.

[18] Fedorkova A., Orinakova R., Orinak A., Heile A., Wiernhofer H.D., Arlinghaus H.F. (2011). Electrochemical and TOF-SIMS investigations of PPy/PEG-modified LiFePO_4_ composite electrodes for Li-ion batteries. Solid State Sci..

[19] Fedorkova A., Orinakova R., Orinak A., Talian I., Heile A., Wiemhofer H.D., Kaniansky D., Arlinghaus H.F. (2010). PPy doped PEG conducting polymer films synthesized on LiFePO_4_ particles. J. Power Sources.

[20] Sharma R.K., Rastogi A.C., Desu S.B. (2008). Pulse polymerized polypyrrole electrodes for high energy density electrochemical supercapacitor. Electrochem. Commun..

[21] Sun J.F., Huang Y., Fu C.X., Wang Z.Y., Huang Y., Zhu M.S., Zhi C.Y., Hu H. (2016). High-performance stretchable yarn supercapacitor based on PPy@CNTs@urethane elastic fiber core spun yarn. Nano Energy.

[22] Hu J.Y., Zhang X.F., Li G.H., Yang X.D., Ding X. (2016). Electrical properties of Ppy-coated conductive fabrics for human joint motion monitoring. Autex Res. J..

[23] Li M.F.F., Li H.Y., Zhong W.B., Zhao Q.H., Wang D. (2014). Stretchable conductive polypyrrole/polyurethane (PPy/PU) strain sensor with netlike microcracks for human breath detection. ACS Appl. Mater. Interface.

[24] Atobe M., Tsuji H., Asami R., Fuchigami T. (2006). A study on doping-undoping properties of polypyrrole films electropolymerized under ultrasonication. J. Electrochem. Soc..

[25] Yang J.J., Di S.C., Blawert C., Lamaka S.V., Wang L.Q., Fu B.L., Jiang P.L., Wang L., Zheludkevich M.L. (2018). Enhanced wear performance of hybrid epoxy-ceramic coatings on magnesium substrates. ACS Appl. Mater. Interface.

[26] Lenz D.M., Delamar M., Ferreira C.A. (2003). Application of polypyrrole/TiO2 composite films as corrosion protection of mild steel. J. Electroanal. Chem..

[27] Jamadade S., Jadhav S.V., Puri V. (2011). Electromagnetic reflection, shielding and conductivity of polypyrrole thin film electropolymerized in P-Tulensulfonic acid. J. Non-Cryst. Solids.

[28] Olad A., Shakoori S. (2018). Electromagnetic interference attenuation and shielding effect of quaternary Epoxy-PPy/Fe_3_O_4_-ZnO nanocomposite as a broad band microwave-absorber. J. Magn. Magn. Mater..

[29] Zhao H., Hou L., Lu Y.X. (2016). Electromagnetic shielding effectiveness and serviceability of the multilayer structured cuprammonium fabric/polypyrrole/copper (CF/PPy/Cu) composite. Chem. Eng. J..

[30] Foroughi J., Spinks G.M., Wallace G.G. (2009). Effect of synthesis conditions on the properties of wet spun polypyrrole fibres. Synth. Met..

[31] Esfahani M.I.M., Taghinedjad S., Mottaghitalab V., Narimani R., Parnianpour M. (2016). Novel printed body worn sensor for measuring the human movement orientation. Sens. Rev..

[32] Chen Y.X., Cai K.F., Liu C.C., Song H.J., Yang X.W. (2017). High-Performance and breathable polypyrrole coated air-laid paper for flexible all-solid-state supercapacitors. Adv. Energy Mater..

[33] Mosnakova K., Chehimi M.M., Fedorko P., Omastova M. (2013). Polyamide grafted with polypyrrole: Formation, properties, and stability. Chem. Pap..

[34] Hu W.H., Li C.M., Cui X.Q., Dong H., Zhou Q. (2007). In situ studies of protein adsorptions on poly (pyrrole-co-pyrrole propylic acid) film by electrochemical surface plasmon resonance. Langmuir.

[35] Lv J.C., Zhou P.W., Zhang L.P., Zhong Y., Sui X.F., Wang B.J., Chen Z.Z., Xu H., Mao Z.P. (2019). High-performance textile electrodes for wearable electronics obtained by an improved in situ polymerization method. Chem. Eng. J..

[36] Kim I., Cho G. (2018). Polyurethane nanofiber strain sensors via in situ polymerization of polypyrrole and application to monitoring joint flexion. Smart Mater. Struct..

[37] Wu J., Zhou D., Too C.O., Wallace G.G. (2005). Conducting polymer coated lycra. Synth. Met..

[38] Hu J.Y., Zhou S.W., Shi J.H., Zhang H.L., Zhu F., Yang X.D. (2017). Determinants of electrical resistance change of in situ PPy-polymerized stretch plain woven fabric under uniaxial tensile strain. J. Text. Inst..

[39] Zhao Y.M., Zhai Q.F., Dong D.S., An T.C., Gong S., Shi Q.Q., Cheng W.L. (2019). A highly stretchable and strain-insensitive fiber-based wearable electrochemical biosensor to monitor glucose in the sweat. Anal. Chem..

[40] Zhao Y.M., Dong D.S., Gong S., Brassart L., Wang Y., An T., Cheng W.L. (2019). A moss-inspired electroless gold-coating strategy toward stretchable fiber conductors by dry spinning. Adv. Electron. Mater..

[41] Gong S., Schwalb W., Wang Y.W., Chen Y., Tang Y., Si J., Shirinzadeh B., Cheng W.L. (2014). A wearable and highly sensitive pressure sensor with ultrathin gold nanowires. Nat. Commun..

