# Supplementary material for: Preparing Polypyrrole-Coated Stretchable Textile via Low-Temperature Interfacial Polymerization for Highly Sensitive Strain Sensor
Source: Micromachines (Basel). 2019 Nov 17;10(11):788. doi: 10.3390/mi10110788 (PMC6915628; doi:10.3390/mi10110788)
Supplement: Supplementary file 1 [file micromachines-10-00788-s001.pdf]

Supplementary Materials

Preparing Polypyrrole-Coated Stretchable Textile via Low-Temperature Interfacial Polymerization for Highly Sensitive Strain Sensor

Xiaodie Chen, Bintian Li, Yan Qiao and Zhisong Lu

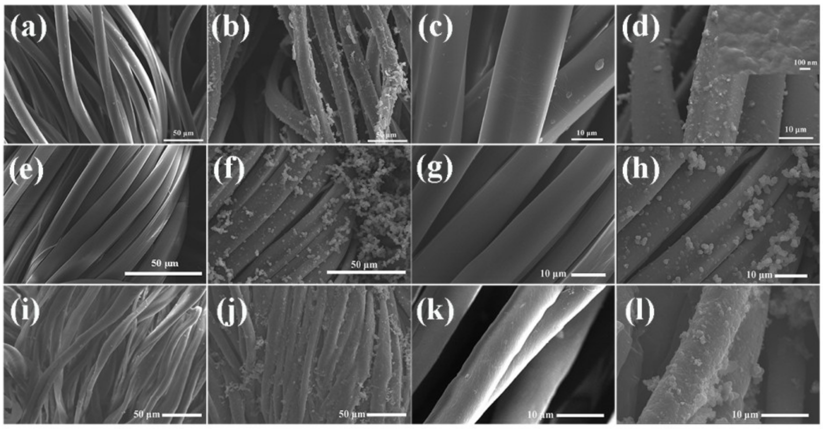

Figure S1. SEM images of pristine nylon/lycra, silk and cotton fabrics at low (a,e,i) and high magnifications (c,g,k); SEM images of PPy-caoted nylon/lycra, silk and cotton fabrics at low (b,f,j) and high magnifications (d,h,l).

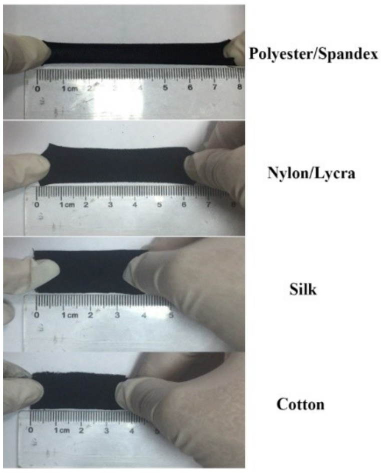

Figure S2. PPy-coated polyester/spandex, nylon/lycra, silk and cotton textiles stretched to the maximum strain.

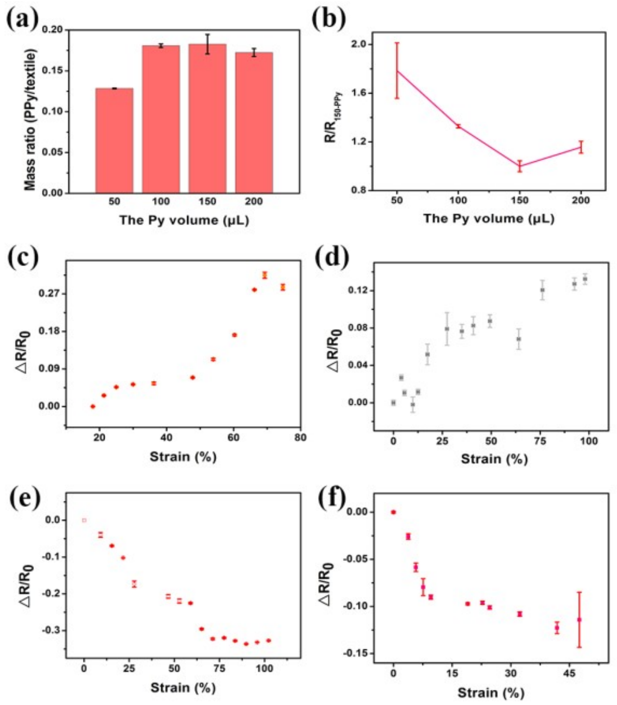

Figure S3. Mass ratios (a) of surface−coated PPy to the textile and resistance ratios (b) of the as-prepared conducting textiles after polymerization with different volume of Py. Relative resistance change ($\Delta R/R_0$) of the as-prepared textiles prepared with 50 μL Py (c), 100 μL Py (d), 150 μL Py (e) and 200 μL Py (f).

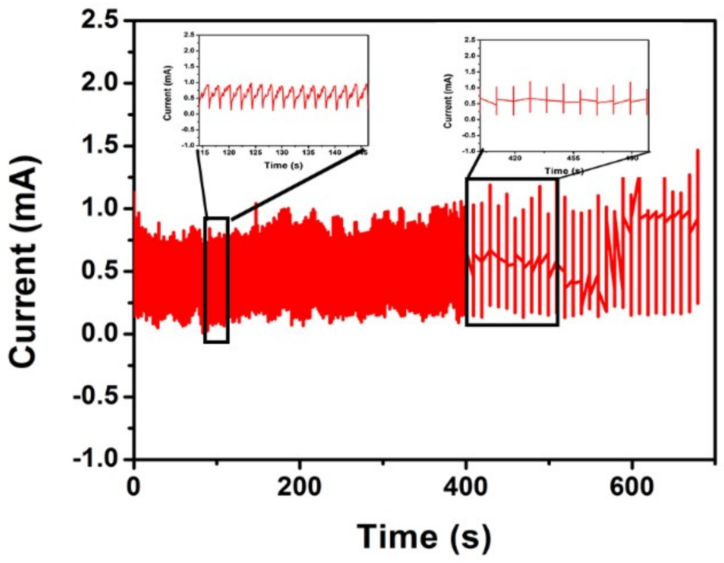

Figure S4. Current responses of a textile-based sensor upon periodic stretching-releasing cycles under a strain of 26%
